# Evaluation of a Commercial Enzyme Linked Immunosorbent Assay (ELISA) for the Determination of the Neurotoxin BMAA in Surface Waters

**DOI:** 10.1371/journal.pone.0065260

**Published:** 2013-06-07

**Authors:** Elisabeth J. Faassen, Wendy Beekman, Miquel Lürling

**Affiliations:** 1 Department of Environmental Sciences, Wageningen University, Wageningen, The Netherlands; 2 Department of Aquatic Ecology, NIOO-KNAW, Wageningen, The Netherlands; University of New South Wales, Australia

## Abstract

The neurotoxin *β*-N-methylamino-L-alanine (BMAA) is suspected to play a role in Alzheimer’s disease, Parkinson’s disease and amyotrophic lateral sclerosis. Because BMAA seems to be produced by cyanobacteria, surface waters are screened for BMAA. However, reliable analysis of BMAA requires specialized and expensive equipment. In 2012, a commercial enzyme-linked immunosorbent assay (ELISA) for determination of BMAA in surface waters was released. This kit could enable fast and relatively cheap screening of surface waters for BMAA. The objective of this study was to determine whether the BMAA ELISA kit was suitable for the determination of BMAA concentrations in surface waters. We hypothesised that the recovery of spiked samples was close to 100% and that the results of unspiked sample analysis were comparable between ELISA and liquid chromatography tandem mass spectrometry (LC-MS/MS) analysis. However, we found that recovery was higher than 100% in most spiked samples, highest determined recovery was over 400%. Furthermore, the ELISA gave a positive signal for nearly each tested sample while no BMAA could be detected by LC-MS/MS. We therefore conclude that in its current state, the kit is not suitable for screening surface waters for BMAA.

## Introduction

The neurotoxin *β*-N-methylamino-L-alanine (BMAA) was discovered in 1967 in cycad seeds from the island of Guam [1] and is suspected to play a role in the neurodegenerative diseases Alzheimer’s disease, Parkinson’s disease and amyotrophic lateral sclerosis [2]. Although there is proof of the neurotoxic effect of BMAA on cellular and animal level, the role of BMAA in the etiology of these neurodegenerative diseases still needs further establishment [3]. Nevertheless, possible pathways of human exposure to BMAA are at present being investigated. After it was reported that BMAA was present in the cyanobacteria that live in symbiosis with the cycads on Guam [4], free living cyanobacteria were screened for BMAA. Initially, BMAA was detected in nearly all tested cyanobacterial species [5]–[7], while some later studies found lower concentrations of BMAA [8], found BMAA only in some samples [9] or did not detect BMAA at all (e.g. [10], [11]). At present, the cause of these differences in BMAA concentrations in cyanobacteria has not been identified yet, although it is very probable that studies that have used the unselective HPLC-FLD (e.g. [5], [6], [12]) have misidentified BMAA and/or overestimated its concentrations [13].

The analytical methods used for unambiguous identification of BMAA in the aquatic ecosystem should be sensitive, selective and robust [13], [14]. Methods based on tandem mass spectrometry like liquid chromatography coupled to tandem mass-spectrometry (LC-MS/MS) meet these requirements, but these types of analysis require rather specialized and expensive equipment. In 2012, a commercial enzyme-linked immunosorbent assay (ELISA) was released. The advantage of such an assay is that a rapid screening of multiple samples can be performed at relatively low costs and with relatively inexpensive equipment. The samples that give a positive signal in the ELISA then need to be further analysed by a more selective analytical method, but if the ELISA works well, time and money can be saved because the amount of samples for more specialized analysis is reduced.

The objective of our study was to determine whether the BMAA ELISA kit was suitable for the determination of BMAA concentrations in surface waters. We performed some basic tests, determined recovery of the ELISA kit in five different samples that were spiked with BMAA and we analysed unspiked water samples from different origin by ELISA and a validated LC-MS/MS method [13]. Because to our knowledge no BMAA was yet detected in untreated (i.e. not extracted or hydrolysed) water, we also included cyanobacterial extracts and hydrolysates in the experiment. We hypothesised that the recovery of the ELISA kit was close to 100% for most tested samples and that the results of the ELISA and the LC-MS/MS analysis were comparable. However, ELISA showed unexplainable deviations in the calibration curve, recoveries were higher than 100% in most spiked samples and nearly each tested sample gave a positive signal in ELISA while no BMAA could be detected by LC-MS/MS. We therefore conclude that the kit is not suitable for screening of surface waters for BMAA.

## Materials and Methods

The ELISA kits were purchased from Abraxis and are based on direct competition: BMAA competes with a BMAA-horseradish peroxidase analogue for binding sites of the rabbit anti-BMAA antibodies in solution. The BMAA antibodies are bound by a goat anti-rabbit antibody that is immobilised on the wells of the plate. The addition of a substrate generates a colour reaction that is inversely proportional to the amount of BMAA present in the sample.

In this study, we tested nine plates. First, the response of the calibration standards provided with the kit was compared to the response of calibration standards prepared in water and in sample diluent (also provided with the kit). Next, as no pH range was given in the manufacturer’s instructions, we determined the response of a BMAA standard at a pH range of pH 1 to 10. We then determined recovery by spiking samples. Finally, a range of unspiked samples was analysed by ELISA and a validated LC-MS/MS method. The experiment was performed in the period from August to December 2012.

### Calibration curves and pH Series

All water used for sample preparation and analysis was purified with a Q-Pod (Millipore). BMAA calibration standards (BMAA hydrochloride, Sigma Aldrich) were prepared in water and in sample diluent (provided with the kit) directly before analysis. A pH series with 250 µg/l BMAA was constructed in a trichloroacetic acid (TCA) solution (pH range 1.4–3), HCl (pH range 1–5) and NaOH (pH range 7–10). The pH series was analysed in duplicate.

### Sample Collection, Pre-treatment and Storage

All samples except the tap water and the humic acid solutions were collected in various lakes and ponds in The Netherlands (Table 1). Tap water was collected in the laboratory and humic acid (Sigma Aldrich) solutions were prepared in Millipore water in the laboratory. Samples 4 and 7 were collected in a PE bottle and homogenized. A part of sample 4 was filtered over a GF/C filter (Whatman), resulting in sample 5. Sample 6 was collected by pushing a core in lake sediment. From this core, the organic top layer of the sediment was collected, centrifuged and the supernatant was filtered over a GF/C filter and collected. Samples 1–7 were stored at 4°C. Samples 8–10 were taken from ponds and lakes with cyanobacterial blooms and were stored at −20°C. Samples 11–14 were also taken from ponds and lakes with cyanobacterial blooms and were lyophilized before storage at −20°C.

**Table 1:** Sample origin, pre-treatment and storage conditions.

Sample name	Origin	City	Date	Cyanobacterial dominance	Pre-treatment	Storage
1 Tap water	Laboratory	n.a.	Nov 2012	n.a.	None	4°C
2 Humic acid 10 mg/l	Laboratory	n.a.	Nov 2012	n.a.	None	4°C
3 Humic acid 100 mg/l	Laboratory	n.a.	Nov 2012	n.a.	None	4°C
4 No bloom unfiltered	Campus pond 1	Wageningen	Nov 2012	None	None	4°C
5 No bloom filtered	Campus pond 1	Wageningen	Nov 2012	None	Filtration	4°C
6 Sediment water	Campus pond 2	Wageningen	Nov 2012	None	Centrifugation and filtration	4°C
7 Brackish	De Veste	Breskens	Nov 2012	None	None	4°C
8 *Pl. rub.* bloom 1	Lake De Kuil	Prinsenbeek	Nov 2010	*Planktothrix rubescens*	None	−20°C
9 *Glo. ech.* bloom	Kralingse Plas	Rotterdam	July 2012	*Gloeotrichia echinulata*	None	−20°C
10 *Micr.* bloom 1	Urban pond	Dongen	June 2010	*Microcystis*	None	−20°C
11 *Ana.* bloom	Campus pond 3	Wageningen	June 2008	*Anabaena*	Lyophilisation	−20°C
12 *Pl. rub.* bloom 2	Wuurdse Plas	Elst	April 2009	*Planktothrix rubescens*	Lyophilisation	−20°C
13 *Aph.* bloom	Lake De Kuil	Prinsenbeek	Oct 2009	*Aphanizomenon*	Lyophilisation	−20°C
14 *Micr.* bloom 2	Gooimeer	Almere	Sep 2009	*Microcystis*	Lyophilisation	−20°C

n.a.: not applicable.

Dominant cyanobacterial species were identified by light microscopy. Chlorophyll-*a* was determined in sample 1–6 by Phyto-PAM (Walz), only sample 4 contained detectable amounts (13 µg/l) of cyanobacterial chlorophyll-*a*. All water samples were fresh, except for sample 7, which had an electric conductivity of 9.3 mS/cm.

No permission was required for sample collection. Samples 4, 5, 6 and 11 were collected from ponds on the campus of Wageningen University, which is private property. As employees of this university, we were allowed to enter the campus freely and to take samples for scientific research. Samples 7–10 and 12–14 were collected from lakes and ponds that were publicly accessible, which is allowed in The Netherlands. Sampling did not involve endangered or protected species and was compliant with the Dutch Flora and Fauna Act.

### Sample Preparation for ELISA Analysis

Directly before analysis, particles were removed from sample 8 by centrifugation and subsequent filtration over a GF/C filter. Sample 10 was also filtered over a GF/C filter and sample 9 was filtered in a tube with a 0.2 µm cellulose acetate filter (Grace Davison Discovery Science) at 16000*g.

Samples 11–14 were extracted in triplicate to release free BMAA from the cyanobacterial cells. 5 mg of sample was extracted at room temperature in the dark for two hours in 300 µl 0.1 N TCA. After the extraction, the sample was centrifuged and the supernatant was transferred. 300 µl 0.1 N TCA was then again added to the pellet and after vortexing and centrifugation the supernatant was pooled with the first supernatant. The pooled supernatant was lyophilized and then dissolved in 600 µl of water brought to pH 7 by NaOH.

The same samples were also hydrolysed in triplicate to determine total BMAA concentration. 1 mg of sample was hydrolyzed in an hydrolysis/derivatization workstation (Eldex), using 6 N HCl liquid hydrolysis for 20 hours at 105°C in the absence of oxygen. Hydrolysates were dried under vacuum and subsequently dissolved in 500 µl water that was brought to pH 7 with NaOH. Both fractions were diluted 5 and 10 times in water with pH 7.

pH of all prepared samples was determined with a paper indicator strip (pH-Fix 0–14, Machery-Nagel).

### Recovery Determination

Samples 1 and 4–6 were used for recovery determination. For this, they were spiked with BMAA to a concentration of 100 µg/l. Unspiked samples were also analysed and recovery (%) was determined as
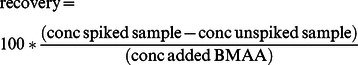
(1)


Extracts and hydrolysates of sample 11 were also used for recovery determination. Extracts and hydrolysates were prepared in sixfold and were dissolved in sample diluent. Both fractions were diluted 5, 10, 100 and 1000 times in sample diluent. Of the undiluted extract/hydrolysate and each dilution, three replicates were spiked to a BMAA concentration of 250 µg/l, while the other three replicates remained unspiked. Recovery was calculated for each dilution with equation 1. As the use of sample diluent gave problems in the recovery determination of sample 11 (see Results), recovery was also determined as described above, but the samples were dissolved in and diluted with water of pH 7.

### ELISA Procedure

ELISA kits were stored at 6°C before analysis and were used before the expiration date. The assay was initially performed according to the instructions of the manufacturer:

- 100 µl of standard solution or sample was added to the wells
- 50 µl of enzyme conjugate solution was added with a multichannel pipette
- 50 µl of antibody solution was added with a multichannel pipette
- The plate was covered with parafilm and the plate was mixed by circular movements for 30 s on the bench top
- The plate was incubated for 90 min at room temperature in the dark
- The plate was washed four times with diluted washing solution (applied with a spray flask) and tapped dry
- 150 µl of substrate solution was added with a multichannel pipette
- The plate was covered with parafilm and the plate was mixed by circular movements for 30 s on the bench top
- The plate was incubated for 30 min at room temperature in the dark
- 100 µl stop solution was added with a multichannel pipette
- Within 15 minutes after the addition of stop solution, absorbance was read at 450 nm on a MTP reader (Synergy HT, BIOTEK).

After the first tests, we added an extra washing step with deionized water after washing with buffer solution. Furthermore, after a few tests, we replaced the sample diluent provided with the kit with water that was brought to pH 7 with NaOH for dissolving and diluting samples.

The calibration curve was constructed by fitting the equation
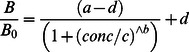
(2)in Sigmaplot 12.0 (Systat Software Inc.), where *B* is the absorption of the calibration standard, *B_0_* is the average absorption of the blank (0 µg/l BMAA) and *conc* is the concentration of the calibration standard. Parameters *a*, *b*, *c* and *d* were estimated. Samples were quantified by comparing the absorption of the sample to the absorption of the calibration curve. Samples with a signal below the signal of the lowest calibration standard (5 µg/l) were reported as not detected.

All samples except the extracts and hydrolysates were analysed in triplicate. The extracts and hydrolysates were already prepared in triplicate, so each replicate was analysed once.

### LC-MS/MS Analysis

Three series of ELISA calibration standards from different lots and samples 1–10 were prepared for underivatised LC-MS/MS analysis. Samples 1–10 were prepared in triplicate. The samples were prepared by adding 10 µl of a 10 mg/l D_3_BMAA solution (internal standard) in 20 mM HCl and 640 µl acetonitrile with 0.15% formic acid to 350 µl sample.

LC-MS/MS analysis was performed according to Faassen *et al.* [13] on an Agilent 1200 LC and an Agilent 6410A QQQ. Compounds were separated on a 2.1×150 mm, 5 µm diameter ZIC®-HILIC column (Sequant) with a Direct-Connect™ Filter (Grace Alltech). Mobile phases were acetonitrile with 0.1% formic acid (v:v, eluent A) and Millipore water with 0.1% formic acid (v:v, eluent B). Flow rate was 0.4 ml/min, injection volume 5 µl and column temperature 40°C. The following gradient was applied: 0–2 min 5% B; 2–4 min linear increase to 35% B; 4–8 min linear increase to 45% B; 8–17 min 45% B; 17–23 min 5% B. Fragmentor voltage was 50 V and both quadrupoles were operated in unit mode. BMAA was detected by the transitions *m/z* 119.1 to *m/z* 102.1 at 4 V collision energy, *m/z 88* and *m/z* 76 (both 8 V). Ratio of both qualifiers *m/z* 88 and *m/z* 76 to quantifier *m/z* 102.1 was 21%. D_3_BMAA was detected by the transitions *m/z* 122.1 to *m/z* 105.1 (4 V), *m/z 88* and *m/z* 76 (both 8 V). Ratio of qualifier *m/z* 88 to quantifier *m/z* 105.1 was 22%, ratio of *m/z* 76 to *m/z* 105.1 was 37%. Calibration standards contained BMAA and D_3_BMAA and were prepared in 65% acetonitrile, 35% Millipore water and 0.1% formic acid (v:v:v). BMAA concentrations in samples were determined by correcting the response of BMAA for the response of D_3_BMAA.

Samples 11–14 were not analysed by LC-MS/MS in this study because they had already been analysed by LC-MS/MS previously [13].

## Results

### Assay Adjustment

According to the manufacturer’s instructions, the plate needed to be washed with the provided washing buffer solution after the first incubation and then patted dry before the substrate solution was added. However, when this protocol was followed, lather remained in the wells, leading to large variation between replicates. We therefore added an extra washing step: after washing with buffer, the plates were washed four times with deionized water and then patted dry. When this procedure was followed, no lather remained on the plate.

### Variation within Replicates

Incidentally, a well gave a value that deviated strongly from the other two replicates without apparent reason. This happened both in the calibration curves and in the samples. Even when the person performing the test was continuously supervised by another person and no mistakes, bubbles or inaccuracies were observed while the test was carried out, these outliers kept occurring. In this study, obvious outliers were not used in the calculation of the calibration curves, but no outliers were omitted from the results.

### Response Standards

**Figure 1:**
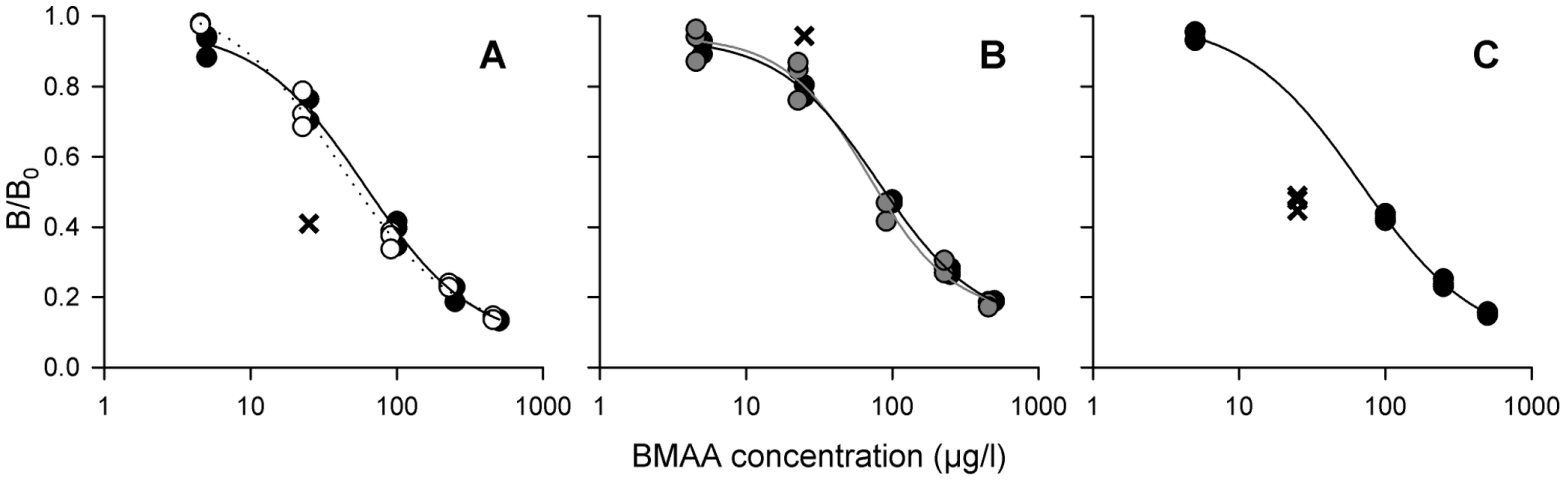
Calibration curves of three of the ELISA plates used in this study. Calibration standards provided with the kit are shown in black circles and solid black lines, calibration standards in water are shown in white circles and dotted black lines and calibration standards in sample diluent are shown in grey circles and grey solid lines. Outliers that are omitted from the calibration curve are shown as black crosses, all outliers belong to the standards from the kit.

The calibration curve of the kit was S-shaped when the horizontal axis was plotted on a logarithmic scale. On three plates, the 25 µg/l standard provided with the kit showed large variation (e.g. Figure 1A and B). On three other plates, this standard gave an absorption close to that of the 100 µg/l standard (Figure 1C). The calibration standards used on one of these latter plates were analysed by LC-MS/MS and according to this analysis, the 25 µg/l standard contained the assigned concentration.

The response of the standards provided with the kit was similar to the response of calibration standards prepared in water and in sample diluent (Figure 1A and B). BMAA standards dissolved in acidic (TCA and HCl) and basic (NaOH) solutions ranging from pH 2.7 to 10 also gave similar results as the calibration standards provided with the kit. Below pH 2.7, their response was higher than that of the kit’s standards, irrespective of whether a TCA or HCl solution was used. Therefore only samples with a pH higher than 3 were analysed in the following experiments.

### Recovery Spiked Samples

**Figure 2:**
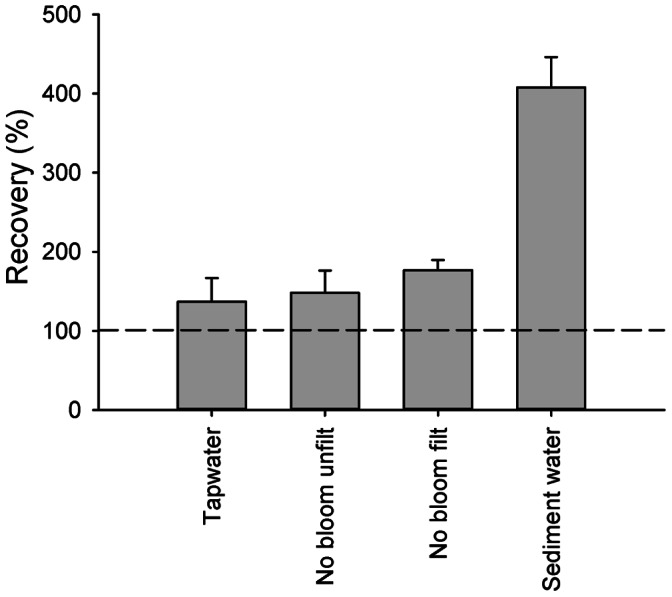
Recovery of spiked samples without cyanobacterial blooms. **Error bars represent one SD, n = 3.**

Recovery was determined in four samples without cyanobacterial dominance by addition of BMAA. For all four samples, recovery was higher than 100%, recovery of the filtered sediment water was highest (408%, Figure 2). pH of these samples was between 7 and 8.

**Figure 3:**
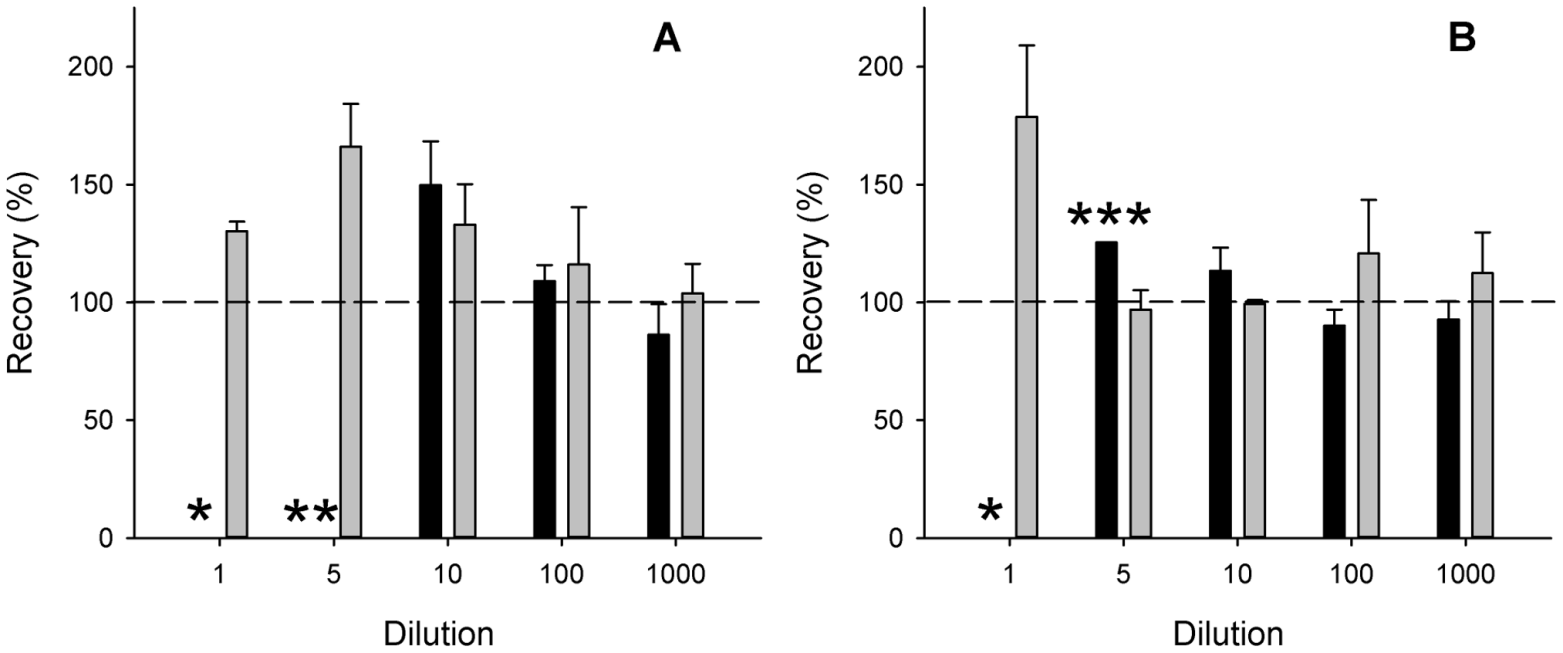
Recovery of spiked extracts (black bars) and hydrolysates (grey bars) of sample 11. In panel A, sample diluent was used as solvent and diluent, in panel B, water brought to pH 7 was used. Error bars represent one SD, n = 3. *: sample not analysed due to too low pH, **: signal of all replicates above calibration curve, which corresponds to recovery >200%, ***: signal of one replicate above calibration curve, bar represents average of other two replicates.

Recovery of extracted and hydrolysed samples was determined in sample 11, a surface water with an *Anabaena* bloom. First, the extracts and hydrolysates were dissolved in and diluted with the sample diluent that was provided with the test. At low dilutions, recovery was higher than 100%. Only when diluted 100 and 1000 times, recovery was close to 100% (Figure 3A). The pH of the undiluted extract was lower than 2 and this sample could therefore not be analysed.

The results of the unspiked samples that were used for the recovery determination showed inconsistencies between replicates and between different dilutions of the same replicate (Tables 2 and 3). As repetition of this part of the experiment (including renewed sample workup) did not give better results, we repeated the experiment again, but then we dissolved and diluted samples in water that was brought to pH 7 with NaOH instead of in sample diluent. Recovery of extracts that were dissolved in water with pH 7 was close to 100% when diluted at least 10 times, while hydrolysates had to be diluted at least 5 times (Figure 3B). The unspiked samples now gave consistent results between replicates and between dilutions (Tables 2 and 3).

**Table 2:** BMAA concentration as determined by ELISA and LOD* (both expressed as µg/g DW) for unspiked extracts of sample 11 in two different solvents.

		Solvent: sample diluent			Solvent: water pH 7		
Dilution	LOD	Replicate A	Replicate B	Replicate C	Replicate D	Replicate E	Replicate F
1	0.6	n.a.	n.a.	n.a.	n.a.	n.a.	n.a.
5	3	14.8	a.c.	a.c.	17.6	25.6	31.0
10	6	n.d.	40.7	17.8	18.2	19.5	19.5
100	60	n.d.	n.d.	76.4	n.d.	n.d.	n.d.
1000	600	n.d.	n.d.	n.d.	n.d.	n.d.	n.d.

* LOD: limit of detection, n.a.: not analysed, sample pH too low, a.c.: above calibration curve (equivalent to >300 µg/g BMAA in sample), n.d.: not detected.

**Table 3:** BMAA concentration by ELISA and LOD* (both expressed as µg/g DW) for unspiked hydrolysates of sample 11 in two different solvents.

		Solvent: sample diluent			Solvent: water pH 7		
Dilution	LOD	Replicate G	Replicate H	Replicate I	Replicate J	Replicate K	Replicate L
1	2.5	35.6	32.8	39.9	37.8	33.7	32.8
5	12.5	29.9	n.d.	78.2	35.3	40.9	39.4
10	25	n.d.	n.d.	99.4	39.7	30.5	49.3
100	250	352.4	n.d.	n.d.	n.d.	n.d.	n.d.
1000	2500	n.d.	n.d.	n.d.	n.d.	n.d.	n.d.

* LOD: limit of detection, n.d.: not detected.

### Response in Unspiked Samples

The response of the ELISA for unspiked water samples is shown in Table 4 (untreated and filtered water samples) and Table 5 (extracted and hydrolysed water samples). Nearly all samples tested positive for BMAA, the ELISA did only not detect BMAA in some replicates of the filtered ‘No bloom’ sample, the sediment water sample and the 10 mg/l humic acid solution. According to the ELISA, samples 9 and 10, which are filtered samples of lakes with a cyanobacterial bloom, contained over 200 µg/l BMAA. Tap water and the humic acid solutions that were prepared in the lab also tested positive for BMAA. All cyanobacterial extracts and hydrolysates were positive for BMAA, in each sample the concentration of total BMAA was higher than that of free BMAA. No BMAA was detected by ELISA in the blanks (purified water and sample diluent).

**Table 4:** BMAA concentrations (µg/l) in untreated and filtered samples as determined by ELISA.

Sample	Treatment	Average	SD	n
1 Tap water	None	12.5	6.1	3
2 Humic acid 10 mg/l	None	16.8	–	2*
3 Humic acid 100 mg/l	None	11.8	10.7	3
4 ‘No bloom’	None	10.7	2.4	3
5 ‘No bloom’	Filtrated	10.8	–	1**
6 Sediment water	Filtrated	16.5	–	1**
7 Brackish	None	19.9	8.4	3
8 *Pl. rub.* Bloom 1	Filtrated	59.2	3.7	3
9 *Glo. ech.* bloom	Filtrated	228.9	14.8	3
10 *Micr.* Bloom 1	Filtrated	298.2	42.5	3

* 1 replicate not detected,

** 2 replicates not detected.

**Table 5:** BMAA concentrations (µg/g DW) in extracted and hydrolysed water samples with cyanobacterial blooms as determined by ELISA.

Sample	Treatment	Average	SD	n
11 *Ana.* bloom	Extraction	19.1	0.7	3
11 *Ana.* bloom	Hydrolysis	39.8	9.4	3
12 *Pl. rub.* bloom 2	Extraction	30.3	4.6	3
12 *Pl. rub.* bloom 2	Hydrolysis	50.1	3.0	3
13 *Aph.* bloom	Extraction	10.5	3.7	3
13 *Aph.* bloom	Hydrolysis	85.3	11.3	3
14 *Micr.* Bloom 2	Extraction	13.8	3.7	3
14 *Micr.* Bloom 2	Hydrolysis	84.4	28.6	3

Concentrations are calculated from the 10 times diluted samples.

No BMAA was detected in any of the samples by LC-MS/MS analysis in this study, neither was it detected in samples 11–14 that had been analysed by LC-MS/MS previously (field scums in table 4 of ref [13]).

## Discussion

### Test Procedure and Application Range

Before starting the final experiments on samples, we adjusted the test protocol at two points: we added an extra washing step with deionized water and we used water brought to pH 7 for dilution of sample extracts and hydrolysates instead of the provided sample diluent. These adjustments made our results more reproducible and consistent. We expect that these changes in the protocol did not have a negative impact on the performance of the test. The plates were washed before the addition of colour substrate, the extra washing step with deionized water could therefore have influenced the colour reaction. However, if such an effect occurred, it has likely been equal for the calibration curves and the samples, so quantification of the samples would not be affected. We also do not expect a negative effect of the use of water for sample dilution as the kit is designed for testing water samples and because the response of calibration standards in water and sample diluents is similar (Figure 1). It was however surprising that the use of Millipore water brought to pH 7 gave better results than the diluent provided with the kit, as the latter consisted of distilled water according to the manufacturer.

The calibration curve of the ELISA is S-shaped, with the steepest part of the curve between 25 µg/l and 250 µg/l. Quantification in this part of the curve is most precise, below 25 µg/l and above 250 µg/l, small changes in absorbance result in relatively large variations in calculated concentrations. The manufacturer reports a level of quantification of 4 µg/l and an upper limit of 500 µg/l. Because the absorbance of the 5 µg/l standard sometimes was close to that of the blank (B/B_0_ close to 1.0, Figure 1), we used a more conservative limit of detection and quantification of 5 µg/l in this study. On three plates, the 25 µg/l standard gave a signal that strongly deviated from the expected calibration curve. This was not caused by a too high BMAA concentration in these standards, as LC-MS/MS analysis confirmed that the calibration standards used on one of these three plates indeed contained the expected concentration. We therefore expect that the problem lied in the wells of the plate, or in an impurity in the calibration standard that interfered during ELISA but not during LC-MS/MS analysis.

### Recovery of Spiked Samples

The recovery of the spiked samples without cyanobacterial blooms was between 137% and 403%. As the pH of these samples was clearly above the critical threshold of 2.7, this overestimation could not be attributed to acidity of the samples. Also the possible presence of BMAA in these samples could not have caused this overestimation, as the concentrations that were determined in the unspiked samples were subtracted from the concentrations in the spiked samples. The recoveries of extracts and hydrolysates of a pond with an *Anabaena* bloom were also higher than 100% at the lowest dilutions. However, the recoveries of the more diluted samples were close to 100%. The mechanism behind these overestimations in spiked samples will be discussed below.

### BMAA Concentration in Unspiked Samples

The ELISA detected BMAA in every tested sample, although in three cases not in every replicate. No BMAA was detected by underivatised LC-MS/MS analysis in any of the samples, even though nearly all the concentrations as determined by ELISA are above the detection limit of the LC-MS/MS method [13]. LC-MS/MS analysis is considered a reliable method for BMAA detection in surface water [13], [14], although some issues have been raised against underivatised LC-MS/MS analysis [15]. However, as we think that the arguments raised by this group are refutable because we used deuterated BMAA as an internal standard [16], we consider the results of LC-MS/MS analysis reliable and therefore assume the ELISA results to be false positives.

### Interfering Compounds in ELISA

As the ELISA gave false positive results and elevated recoveries for most samples, it is likely that components in the samples have interfered. Because purified water and sample diluent contained no BMAA according to ELISA and gave accurate results when BMAA was added (Figure 1A and B), the problems seemed not to be caused by these solvents. One mechanism that could cause false positives and overestimation in an ELISA test is cross-reactivity: the antibody in the test does not only react with the analyte (in this case BMAA), but also with other molecules in the sample. According to the manufacturer, the BMAA ELISA shows cross-reactivity with L-cysteine hydrochloride, L-glutamic acid, L-aspartic acid (all 0.2% of BMAA signal), γ-aminobutyric acid (0.02%) and DL-2,4-diaminobutyric acid dihydrochloride (0.01%). As all of these compounds can be present in cyanobacteria (e.g. [9], [17]–[19]), these compounds might indeed have increased the signal. However, cross-reactivity of only these five reported compounds is unlikely to be the only cause of the frequent occurrence of false positives with sometimes high concentrations. It is likely that the test shows cross-reactivity with more compounds. From our experiments we can identify humic acids as likely being cross reactive: a 100 mg/l humic acid solution in purified water gave a BMAA signal corresponding to 11.8 µg/l BMAA (Table 4).

Besides cross-reactivity, other types of interferences seemed to have occurred in our experiments. The elevated recoveries of most spiked samples cannot be explained by cross-reactivity, as the recovery calculation was based on the differences in concentration between spiked and unspiked samples. According to the manufacturer, the kit can be used in a variety of inorganic solutions and in a 10% seawater solution. The electric conductivity of the brackish sample in this study was approximately 20% of that of the neighbouring seawater, so in this sample the seawater might have interfered. However, for the other samples we do not know which mechanisms are responsible for the observed overestimation as it happened in samples that varied greatly in origin and composition. Testing for possible interferences and identifying the underlying mechanisms is a laborious task that is normally carried out during test development and we therefore considered it beyond the scope of this study.

### Conclusions

The objective of this study was to determine whether the evaluated ELISA kit is suitable for determination of BMAA concentrations in surface water. To our opinion, the kit (in its current state) should not be used for this purpose. One problem with the kit is that in one third of the tested cases, no decent calibration curve could be constructed because one standard strongly deviated from the expected line. On all tested plates, outliers occurred that could not be explained by obvious errors or inaccuracies. More importantly, the test gave elevated recoveries for a diversity of spiked samples and gave false positive results in nearly all tested samples. Although the manufacturer states that the test should be used for screening purposes and that additional analytical analysis should be performed to confirm positive results, a nearly 100% score of positives in samples that are unlikely to contain detectable amounts of BMAA makes the test unsuitable for its intended purposes. As a good screening method for BMAA in surface waters can be very useful, we recommend further development of the test.
